# AI Agent-Driven Intelligent Catalog Framework: A Governance-Centered Approach for Cleaning and Normalization of Heterogeneous Industrial Sensor Data

**DOI:** 10.3390/s26113589

**Published:** 2026-06-04

**Authors:** Hongyi Dong, Yimeng Zhang, Yifan Chu, Hailing Zhou, Mingxin Lu, Zuojian Zhou, Xiaoyang Zhou

**Affiliations:** 1 Department of Information Management, Nanjing University, Nanjing 210033, China; hongyixunxundong@163.com (H.D.); zhangyimeng050419@163.com (Y.Z.); rosazhou0530@163.com (H.Z.); mxlu@nju.edu.cn (M.L.); 2 Nanjing University (Suzhou) High-Tech Institute, Suzhou 215123, China; 3 School of Artificial Intelligence and Information Technology, Nanjing University of Chinese Medicine, Nanjing 210033, China; zhouzj@njucm.edu.cn; 4 China Mobile Zijin (Jiangsu) Innovation Research Institute, Nanjing 211899, China

**Keywords:** Industrial Internet of Things, data governance, heterogeneous sensor data, Intelligent Catalog, AI Agent, workflow orchestration

## Abstract

The rapid development of the Industrial Internet of Things (IIoT) generates massive heterogeneous sensor data, complicating data cleaning and normalization. Existing algorithmcentric methods often treat quality issues in isolation and lack unified governance. This paper proposes a governance-centered framework for multi-source industrial sensor data. We introduce an Intelligent Catalog as the semantic governance layer to standardize metadata and achieve semantic alignment before numerical processing. Building upon this, an AI Agent-driven mechanism dynamically orchestrates cleaning and normalization strategies based on real-time data status and heterogeneous features. This framework modularly integrates classical algorithms (e.g., PCA, KPCA, LSTM) without model dependency. Experimental results on public IIoT datasets demonstrate that our framework significantly outperforms baseline methods in normalization consistency, noise robustness, and stability across heterogeneous data. By shifting from an algorithm-centered to a governance-centered paradigm, this approach provides a scalable and adaptive solution for complex industrial sensor data management.

## 1. Introduction

### 1.1. Industrial Sensor Data as a Data Governance Problem

The industrial internet has become the foundational infrastructure of modern industrial systems, enabling real-time monitoring, predictive maintenance, and intelligent decision-making. Industrial sensors deployed in production lines, equipment systems, and energy networks continuously generate vast amounts of time-series data, which are increasingly viewed as vital industrial assets rather than mere byproducts of system operations.

However, despite the immense potential value of industrial sensor data, its reuse and integration still face numerous difficulties, primarily stemming from inherent heterogeneity. Sensors from different manufacturers, operating under diverse working conditions and adopting various collection strategies, exhibit significant differences in data formats, semantics, scales, and quality. As industrial systems continually evolve, this heterogeneity becomes increasingly prominent, persistently challenging downstream analysis and intelligent applications. From a practical perspective, data cleaning and normalization are not merely technical preprocessing steps but crucial components of industrial data governance. Sensor data lacking effective governance will propagate errors throughout the analysis pipeline, weaken model reliability, and ultimately impact industrial decisions. Therefore, industrial sensor data management urgently requires a systematic, adaptive data governance mechanism rather than fragmented algorithmic solutions.

### 1.2. Challenges Posed by Industrial Sensor Data Heterogeneity

The heterogeneity of industrial sensor data manifests on multiple levels.

- Structurally, data may be stored in flat tables, nested structures, or semi-structur-ed formats.
- Semantically, the same physical quantity may be represented by different labels in different systems, while fields with similar names might possess different meanings.
- Statistically, sensor readings exhibit significant differences in value ranges, distribution shapes, noise characteristics, and temporal behaviors.

This multi-level heterogeneity significantly increases the complexity of data cleaning and normalization. Simple normalization methods might achieve alignment on a numerical scale but ignore semantic inconsistencies, leading to misleading results. Conversely, complex machine learning methods, while capable of capturing high-level patterns, are often sensitive to changes in data distribution and require frequent retraining. Therefore, addressing this heterogeneity requires a holistic approach that integrates semantic alignment, adaptive processing strategies, and algorithmic flexibility.

### 1.3. Limitations of the Algorithm-Centered Paradigm

Most existing studies treat data cleaning and normalization as isolated algorithmic problems. Rule-based methods rely on predefined heuristics and expert experience, which are difficult to maintain in dynamic industrial environments. Statistical methods are computationally efficient but lack robustness under non-stationary and non-linear conditions. In industrial contexts, “non-linear conditions” typically refer to scenarios where sensor responses become disproportionate near equipment physical limits, or where the interactions between multiple physical quantities (e.g., the complex coupling between temperature, pressure, and vibration during a fault) cannot be adequately modeled by simple proportional relationships. Consequently, linear statistical tools often fail to capture the true underlying data structures in these dynamic environments. Machine learning and deep learning methods excel in specific tasks like anomaly detection but typically require large amounts of annotated data and are highly coupled with specific data distributions.

More importantly, these algorithm-centered methods usually lack a global data governance perspective. They focus on “how to process data” but rarely answer “how data should be organized, understood, and managed long-term across systems”. This deficiency limits their application in large-scale, multi-source industrial environments.

### 1.4. Research Objectives and Contributions

To overcome these shortcomings, this paper redefines industrial sensor data cleaning and normalization as a governance-oriented process. By introducing an Intelligent Catalog as the semantic foundation and an AI Agent as the decision-making entity, the proposed framework enables adaptive, reusable data governance in heterogeneous sensor environments.

The main contributions of this paper are as follows:

- First, it proposes a governance-centered framework oriented towards multivariate heterogeneous industrial sensor data, unifying semantic alignment and numerical processing. This effectively accomplishes data cleaning and normalization tasks while minimizing semantic loss.
- Second, it designs an Intelligent Catalog mechanism to standardize metadata representation and support dynamic data augmentation. The system innovatively introduces a reference set of common standard data attributes and a data attribute substitution vocabulary, effectively solving the lack of data normalization standards in the industrial sensor field. It also introduces automatic annotation and sample augmentation technologies to transform new processed data into incremental additions to the attribute set and vocabulary, enabling dynamic updates and improving the capability to handle complex situations.
- Third, it introduces an AI Agent-driven mechanism to dynamically orchestrate cleaning and normalization workflows based on data characteristics. Leveraging AI Agent technology and the MCP protocol, the system combines the advantages of LLMs in semantic understanding and information disambiguation for text-type data with the accuracy and robustness of traditional machine learning for numerical data, allowing the system to excel in data cleaning and normalization under complex, real-world industrial conditions.

## 2. Related Work

### 2.1. Data Cleaning and Normalization in Traditional Industrial Systems

Traditional data processing models, such as rule-based cleaning and simple statistical normalization, laid the foundation for early industrial systems [1]. However, their static nature makes them inadequate for modern IIoT environments characterized by high-frequency, multi-modal heterogeneous data [2,3]. Recent research in 2024 and 2025 emphasized the paradigm shift toward adaptive and context-driven preprocessing to manage sensor drift and inconsistent data quality [4]. For instance, Yan et al. (2024) demonstrated that adaptive normalization frameworks significantly outperform static scaling when dealing with non-stationary sensor streams in complex industrial faces [5]. Despite these algorithmic advancements, a persistent challenge remains: these methods typically operate under the assumption that data is already semantically aligned, lacking a systematic metadata governance perspective prior to numerical execution.

### 2.2. Machine Learning Methods for Sensor Data Quality Management

The integration of Machine Learning (ML) and Deep Learning (DL) has revolutionized industrial sensor data correction, showing distinct advantages in handling non-linear noise and anomalies. Recent benchmark studies, such as the DataSense framework (2025) [6], highlight the effectiveness of multi-objective ML pipelines in isolating relevant features and detecting anomalies across heterogeneous IIoT streams. However, traditional deep learning networks often suffer from poor generalization and require extensive retraining when sensor data distributions drift.

To overcome the limitations of static models, the research frontier has rapidly shifted towards autonomous systems powered by Large Language Models (LLMs). Jannell et al. (2025) [7] highlighted the transformative potential of “Agentic LLMs” in industrial environments, demonstrating that AI agents can autonomously use external tools, query databases, and seek consensus in complex, unstructured data scenarios. Furthermore, as noted by Xu et al. (2024) [8], Agentic LLMs can interpret natural language and contextualize heterogeneous metadata in real-time. This structural shift provides a strong theoretical basis for using AI Agents to orchestrate data cleaning strategies dynamically, rather than relying on isolated algorithms.

### 2.3. Explorations in Multi-Sensor Information Fusion

Multi-sensor information fusion offers supplementary strategies for data cleaning by aggregating data from heterogeneous sources to improve system robustness and reduce uncertainty [9]. The recent literature has moved far beyond traditional artificial neural network fusion. Modern optimized frameworks integrate big data analytics with advanced dynamic filtering techniques to enhance state estimation and adaptive noise accommodation in IoT ecosystems [10]. As noted in recent top-tier reviews on multi-sensor data fusion [11], effectively handling and modeling the uncertainty of information is crucial for improving data quality. Nevertheless, while these mathematical fusion techniques manage numerical uncertainty effectively, they inherently struggle to resolve semantic ambiguities across disparate sensor networks without a unified catalog or metadata schema. This gap underscores the critical need for a governance-centered framework that precedes and guides numerical fusion.

In summary, current research on industrial sensor data cleaning and normalization has four core deficiencies:

1. Lack of a unified multi-source heterogeneous data governance framework to resolve semantic conflicts and format differences.
2. Existing algorithms lack flexibility or generalization to adapt to dynamically changing noise types and heterogeneous data features.
3. Algorithms are insufficiently integrated with practical industrial applications, lacking end-to-end architecture verification.
4. Most studies focus on a single sensor or processing step, failing to achieve collaborative governance of multi-source sensor data required for large-scale industrial applications.

### 2.4. Intelligent Catalog Technology

As an effective carrier for multi-source heterogeneous data integration, the core adaptability of Intelligent Catalog technology can provide support to solve the aforementioned challenges. Intelligent Catalogs abandon the static limitations of traditional digital catalogs to form an intelligent, multidimensional, and dynamically interactive data organization carrier [12]. They can integrate multi-dimensional metadata such as parameter specifications, data formats, and sampling features of different sensors to build a three-dimensional “sensor data portrait,” providing a stable reference for data normalization [13]. At the same time, Intelligent Catalogs can connect manageable metadata with executable processing flows, realizing automatic data identification, classification, and preliminary alignment through semantic analysis and knowledge graph technologies [14]. By introducing AI Agents and MCP, the agent can undertake strategy selection and workflow orchestration for the “alignment—cleaning—normalization” phases, flexibly invoking various traditional methods and algorithms [15]. This fusion idea provides a new implementation path for unified governance of multivariate heterogeneous industrial sensor data.

Based on this, this paper intends to use the Intelligent Catalog as the governance hub, with dimensionality reduction methods like PCA/KPCA and time-series models like LSTM as fundamental algorithms [16,17]. At the execution level, an AI Agent is introduced for strategy selection and workflow orchestration, bringing external tools into a unified execution chain via the MCP mechanism to form a traceable “alignment—cleaning—normalization” process. Finally, experiments are conducted on public industrial datasets from *awesome-industrial-datasets* [18] to evaluate indicators such as anomaly detection accuracy, correction error, alignment consistency, and runtime overhead, comparing them against baseline models to verify the scheme’s effectiveness.

## 3. Methodology

### 3.1. Overall Design Philosophy and Workflow

Traditional preprocessing methods usually assume that industrial sensor data have already been aligned in terms of structure, semantics, and units. In real Industrial Internet of Things (IIoT) environments, however, this assumption rarely holds. Sensor streams may differ in attribute names, sampling frequencies, nested schema structures, units of measurement, value ranges, and noise patterns. Therefore, this study reformulates data cleaning and normalization as a governance-centered problem rather than a purely algorithmic preprocessing task.

From the perspective of library and information science, heterogeneous sensor data can be regarded as catalogable digital objects. Their raw column names are equivalent to unstable access points, their metadata fields constitute descriptive elements, and their standardized sensor attributes function as controlled headings or authority records. Accordingly, the proposed framework introduces an Intelligent Catalog to perform semantic authority control before numerical computation. This design ensures that numerical operations are not applied to semantically incompatible variables.

The proposed framework is organized into three tightly coupled layers: (1) the Intelligent Catalog layer, which governs metadata, controlled vocabularies, semantic mapping, and field alignment; (2) the AI Agent decision layer, which analyzes data states and constructs appropriate processing strategies; and (3) the execution layer, which invokes modular numerical tools to perform cleaning, correction, dimensionality reduction, temporal correction, and normalization. The complete workflow is as follows: heterogeneous sensor data are first registered and semantically aligned in the catalog, then summarized into a state vector describing structural, semantic, statistical, and temporal conditions, after which the AI Agent selects or composes a processing policy and dispatches executable tools. Finally, the processed results, selected parameters, and execution traces are written back as governance feedback to support future reuse.

In the open-source prototype, this workflow is implemented as a Python-based pipe-line. The core entry point is IndustrialDataGovernancePipeline in main_pipeline.py. The pipeline receives three inputs: a raw sensor table represented as a pandas.DataFrame, a list of SensorMetadata records, and an optional target dataframe used for cross-source distribution alignment. The pipeline then performs semantic alignment, state perception, AI Agent-based or heuristic workflow planning, modular tool invocation, and fallback processing when an exception occurs. Specifically, _semantic_align() first generates probabilistic semantic labels for each metadata record and renames raw columns into canonical sensor names; perceive_data_state() then computes the missing-value ratio, noise level, and outlier status; build_planning_prompt() combines the semantic mapping and data state into an agent-planning prompt; and the selected processing functions are finally executed on the aligned dataframe. If no LLM or LangChain agent is available, the same pipeline automatically switches to a deterministic heuristic execution flow, thereby preserving reproducibility and avoiding dependence on a single LLM runtime. Further details on the system architecture are shown in Table 1 below.

This layered design ensures that improvements in one layer, such as enhanced semantic alignment capabilities, can benefit the entire system without requiring modifications to specific algorithmic implementations.

### 3.2. Intelligent Catalog Layer

#### 3.2.1. Motivation for Semantic Governance

The heterogeneity of industrial sensor data is rooted not only in numerical differences but also in semantic inconsistencies. For example, the same physical quantity in different systems might use different field names, units of measurement, or contextual semantic representations. If numerical normalization is performed directly without resolving semantic differences, it is highly likely to produce misleading or even erroneous processing results.

Therefore, this paper introduces the Intelligent Catalog as the semantic foundation of the entire framework. Unlike traditional data catalogs mainly used for data discovery, the proposed Intelligent Catalog is explicitly aimed at supporting data cleaning and normalization. It enforces semantic consistency before numerical processing and thus functions as a governance layer for trustworthy sensor-data reuse.

**Figure 1:**
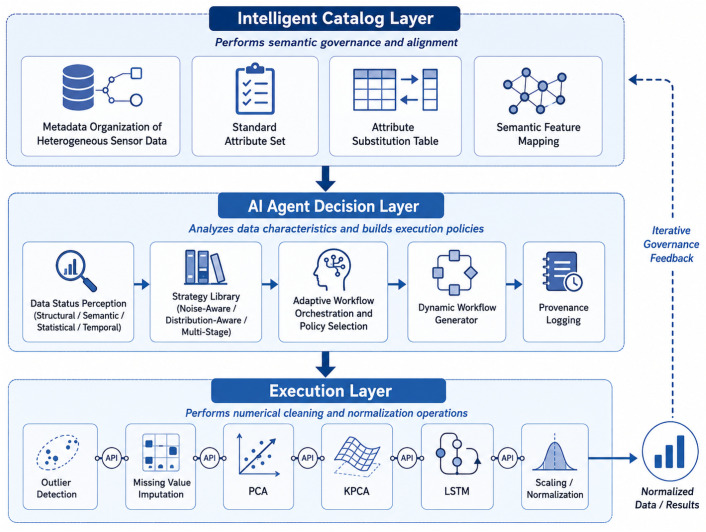
Revised AI Agent-driven framework for heterogeneous sensor data governance and normalization. The framework consists of an Intelligent Catalog layer for semantic governance, an AI Agent decision layer for adaptive workflow orchestration, and an execution layer for modular numerical processing. A feedback loop supports iterative governance.

In implementation, the catalog layer is represented by two components: a metadata schema and a weakly supervised semantic labeling module. The metadata schema is defined by the SensorMetadata data structure, which records the raw field name, mean value, engineering unit, and sampling frequency of each sensor stream. The current standard attribute space is defined by the SensorType enumeration, including TEMPERATURE, PRESSURE, VIBRATION, CURRENT, VOLTAGE, and ABSTAIN. The ABSTAIN label is used when the catalog cannot confidently assign a raw field to an existing standard attribute, thereby preventing forced semantic matching. And the content of the intelligent catalog layer is shown in Figure 1 below.

#### 3.2.2. Construction of Standard Attribute Sets

A core component of the Intelligent Catalog is the standard attribute set, which defines a unified semantic schema for industrial sensor data. Each standard attribute encapsulates a set of key metadata fields, including sensor identifier and type, measured physical quantity, measurement unit and scale, sampling frequency and temporal resolution, expected value range, and contextual constraints.

To support spatial consistency analysis, the schema can be further enriched with spatial and contextual attributes, including deployment coordinates, zone ID, equipment-relative position, and environmental baseline indicators, such as proximity to heat or vibration sources. These attributes allow the system to define semantic neighborhoods for sensors that are physically expected to exhibit correlated or divergent behaviors.

The construction of the standard attribute set combines domain knowledge analysis with empirical observations of public industrial sensor datasets. In the implemented prototype, the initial standard set is operationalized through lightweight semantic classes corresponding to common industrial sensing modalities. For instance, a temperature stream is identified not only by a field name such as temp_main, but also by its engineering unit, typical value range, and sampling behavior. A vibration stream, by contrast, may be identified by terms such as vib, vibration, or accel, combined with a high sampling frequency. This design follows the logic of authority control in information organization: the raw expression of an attribute may vary, but the catalog links variant expressions to a controlled standard heading.

To address the cold-start problem, the Intelligent Catalog employs a knowledge-driven bootstrap mechanism. The initialization process does not rely solely on empirical data distributions. Instead, the standard attribute set is pre-populated using domain expert heuristics and common industrial metadata conventions. Once the first batch of heterogeneous data is ingested, the AI Agent performs an initial structural and semantic audit to map raw streams to the predefined catalog. In the prototype, this audit is implemented through labeling functions and probabilistic label generation. If the Snorkel framework is unavailable, the catalog module automatically falls back to a voting-based labeling mechanism, which ensures that the pipeline remains executable even in a lightweight runtime environment.

#### 3.2.3. Attribute Substitution and Semantic Alignment

To address field-level heterogeneity, this paper introduces an attribute substitution table to record the mapping relationships between heterogeneous field names and standard attributes. This mechanism supports one-to-one, many-to-one, and conditional mappings. For instance, different manufacturers may use labels such as Temp, temperature, Wendu, or temp_main to represent temperature measurements. These variant labels are aligned to the same standard attribute through the catalog.

In the prototype, this semantic alignment is implemented in snorkel_labeler.py and main_pipeline.py. The labeling module defines multiple labeling functions. Some functions use keyword evidence, such as detecting temp, temperature, wendu, or deg in the field name. Others use value-unit constraints, such as assigning a field to temperature when its mean value lies within an expected temperature range and its unit is c or celsius. Pressure is similarly identified through keywords such as press or pressure, as well as units such as mpa, bar, or kpa. Vibration is identified through both naming evidence and high sampling frequency. These labeling functions generate noisy but useful semantic signals, which are then aggregated by a Snorkel label model or, when Snorkel is not available, by a fallback voting strategy.

The semantic alignment process produces a semantic_map, which maps raw field names to canonical names. For example, temp_main may be mapped to temperature, press_line_a to pressure, and vib_motor_1 to vibration. If multiple fields are mapped to the same standard attribute, the pipeline preserves them by adding suffixes such as temperature_2 rather than overwriting them. If no confident label is found, the original field name is retained and the item is treated as a low-confidence catalog entry for later review. This mechanism prevents both semantic loss and accidental merging of distinct sensor streams.

During data ingestion, input sensor data are first matched against the attribute substitution mechanism. If the mapping is successful, the corresponding standard attribute is assigned. If the mapping confidence is insufficient, the item is flagged for governance feedback instead of being forcibly normalized. This process ensures that semantic alignment is completed before numerical cleaning and normalization, significantly reducing the risk of semantic conflicts.

### 3.3. AI Agent Decision Layer

The AI Agent does not directly manipulate raw numerical values. Instead, it functions as an orchestration engine that perceives data states, selects strategies, composes execution workflows, and records provenance information. To remain practical for IIoT scenarios, the framework adopts an offline-orchestration/online-execution paradigm: the AI Agent resides in the control plane and updates governance policies asynchronously, while the data plane executes lightweight numerical tools at run time.

For each incoming dataset or micro-batch, the AI Agent extracts a state vector. Conceptually, the state vector contains four groups of features: structural features, semantic features, statistical features, and temporal features. The current prototype operationalizes these dimensions through a compact but executable set of indicators. Structural quality is represented by the overall missing-value ratio, computed as the number of missing cells divided by the total number of cells. Statistical quality is represented by an outlier flag and a noise-level estimate. The outlier flag is determined by the IQR rule for each numerical column. The noise level is estimated from first-difference volatility: the standard deviation of the first-order difference is divided by the original standard deviation, and the mean ratio is classified as low, medium, or high. Semantic information is represented by the catalog-generated semantic_map, which records the standardized correspondence between raw fields and canonical attributes. Temporal conditions are reflected by volatility and, when necessary, handled by the LSTM-based correction module.

#### Prompt Design for the AI Agent Decision Layer

To make the AI Agent decision process explicit, reproducible, and auditable, this study designs a set of task-specific prompts for the AI Agent decision layer. These prompts correspond to different stages of the decision process, including state summarization, strategy selection, tool planning, semantic constraint checking, distribution alignment decision, temporal correction decision, fallback decision, and provenance logging.

P1: State summarization. The first prompt is used for state summarization. Its purpose is to guide the AI Agent to diagnose the current data quality condition from structural, semantic, statistical, and temporal dimensions. The prompt template is as follows: “You are an industrial sensor data governance agent. Given the semantic mapping and data-state indicators, summarize the current data quality condition from structural, semantic, statistical, and temporal dimensions. Semantic map: {semantic_map}. Missing ratio: {missing_ratio}. Noise level: {noise_level}. Outlier status: {has_outlier}. Target dataframe available: {target_available}.” The input variables include semantic_map, missing_ratio, noise_level, has_outlier, and target_available. The expected output is a diagnostic summary of the current data state.

P2: Strategy selection. The second prompt is used for strategy selection. Its purpose is to help the AI Agent select an appropriate cleaning and normalization strategy based on the diagnosed data state. The prompt template is as follows: “Based on the diagnosed data state, select an appropriate cleaning and normalization strategy. Follow these rules: missing values should be repaired before scaling; outliers and medium/high noise should trigger anomaly correction; cross-source alignment should only be applied to semantically homologous variables; high temporal instability should trigger LSTM-based temporal correction.” The input variables include the data-state summary and the available tools. The expected output is a selected processing strategy with ordered steps.

P3: Tool planning. The third prompt is used for tool planning. Its purpose is to convert the selected strategy into an executable tool sequence. The prompt template is as follows: “Convert the selected strategy into an executable tool sequence. Available tools include KNN imputation, IQR anomaly correction, PCA, KPCA, RBF-MMD distribution normalization, LSTM temporal correction, and min-max normalization. Return only an ordered list of tool names and the reason for each tool.” The input variables include the selected strategy and tool descriptions. The expected output is an ordered tool sequence and its rationale.

P4: Semantic constraint check. The fourth prompt is used for semantic constraint checking before numerical alignment. Its purpose is to prevent the system from aligning sensor variables that represent different physical quantities. The prompt template is as follows: “Before applying any numerical alignment, verify whether the columns to be aligned are semantically homologous. Use the semantic map: {semantic_map}. Do not align variables that represent different physical quantities, such as temperature and pressure.” The input variables include semantic_map and candidate columns. The expected output is a validation result, namely approved, rejected, or requires review.

P5: Distribution alignment decision. The fifth prompt is used to determine whether RBF-MMD distribution normalization should be applied. Its purpose is to ensure that distribution alignment is performed only when the source and target variables are semantically comparable. The prompt template is as follows: “Determine whether RBF-MMD distribution normalization should be applied. Apply it only when a target dataframe is available and when source and target columns share the same canonical attribute. Target dataframe available: {target_available}. Shared columns: {shared_columns}.” The input variables include target_available, shared_columns, and semantic_map. The expected output is a decision on whether to apply RBF-MMD and to which columns.

P6: Temporal correction decision. The sixth prompt is used to determine whether LSTM temporal correction should be applied. Its purpose is to evaluate whether the current sensor stream contains sufficient temporal instability or dependency to justify temporal correction. The prompt template is as follows: “Determine whether LSTM temporal correction should be applied. Use the noise level, volatility estimate, sampling regularity, and temporal dependency indicators. Noise level: {noise_level}. Volatility score: {volatility_score}. Sampling regularity: {sampling_regularity}.” The input variables include noise_level, volatility_score, and sampling_regularity. The expected output is a decision on whether to apply LSTM temporal correction.

P7: Fallback decision. The seventh prompt is used for fallback decision-making. Its purpose is to ensure that the pipeline remains executable when the selected tool sequence fails or when required dependencies are unavailable. The prompt template is as follows: “If the selected tool sequence fails or required dependencies are unavailable, choose a safe fallback workflow. The fallback should preserve executable robustness and avoid semantic damage.” The input variables include the error message, the failed tool, and the current dataframe status. The expected output is a fallback workflow.

P8: Provenance logging. The eighth prompt is used for provenance logging. Its purpose is to generate a structured record of the executed workflow so that the processing procedure can be audited and reused by the Intelligent Catalog. The prompt template is as follows: “Generate a provenance record for the executed workflow. Include the semantic map, data-state vector, selected tools, parameter settings, failed tools if any, fallback operations, and final output status.” The input variables include semantic_map, state_vector, selected_tools, parameters, and execution_logs. The expected output is a structured provenance record for catalog feedback.

The state vector is then evaluated against a decision logic matrix. In the implemented prototype, the AI Agent can operate in two modes. When an LLM and LangChain are available, the system initializes a ZERO_SHOT_REACT_DESCRIPTION agent with a governance prompt. This prompt instructs the agent to choose among noise-aware normalization, distribution-aware normalization, and multi-stage processing. When the LLM is unavailable, the system automatically switches to a deterministic heuristic planner. The heuristic planner follows explicit rules: if the missing ratio is greater than zero, KNN imputation is inserted; if outliers exist or the noise level is medium/high, IQR-based anomaly correction is inserted; if a target dataframe is available, MMD-based distribution normalization is inserted; and if the noise level is high, LSTM-based temporal correction is appended.

This dual-mode design improves reproducibility. The LLM-enabled agent supports flexible reasoning over semantic and statistical conditions, while the heuristic fallback guarantees that the same pipeline can still run under restricted computational or deployment environments. In both modes, the agent does not hard-code a single processing pipeline. Instead, it dynamically constructs an executable sequence based on the observed data state. These indicators and rules are shown in Table 2 below.

Once the strategy is selected, the AI Agent converts it into an executable tool sequence. In the conceptual architecture, this corresponds to an MCP-enabled tool-dispatch layer. In the current prototype, this layer is implemented as a LangChain-compatible tool registry in agent/tools.py. Each tool wrapper has a standardized description and operates on a shared working dataframe. The registered tools include IQR anomaly correction, KNN imputation, PCA dimensionality reduction, MMD distribution normalization, and LSTM time-series correction.

### 3.4. Execution Layer and Modular Algorithm Integration

The execution layer contains modular tools with standardized input–output interfaces. This design decouples decision logic from algorithm implementation and makes the framework extensible. In the current prototype, each processing function accepts a pandas.DataFrame as input and returns a processed pandas.DataFrame as output. Numerical operations are applied only to numeric columns, while non-numeric columns are preserved when applicable. This uniform interface allows the AI Agent or heuristic planner to combine tools without rewriting algorithm-specific code.

The current implementation includes two groups of tools: basic processing tools and advanced processing tools.

First, processing/basic_tools.py implements four basic operations. The IQR anomaly-correction function detects pulse-like outliers by computing the first quartile, third quartile, and interquartile range for each numeric column. Values outside the interval are clipped to the corresponding boundary. This approach is suitable for industrial signals with abnormal spikes while preserving the overall median trend. The KNN imputation function uses KNNImputer with n_neighbors = 5 to fill missing numerical values, which is appropriate when missingness is moderate and variables are correlated. The PCA module first fills remaining missing values with column medians, standardizes numerical columns with StandardScaler, and then performs PCA while retaining the target explained variance ratio, set to 0.95 by default. The min-max normalization module scales numerical columns into a unified range. In addition, the basic fallback flow performs linear interpolation, median filling, and min-max normalization when the full pipeline fails, ensuring operational robustness.

Second, processing/advanced_tools.py implements distribution-aware and temporal correction tools. The MMD distribution-normalization function identifies numeric columns shared by the source and target dataframes and aligns only these shared, semantically comparable variables. It computes RBF-kernel Maximum Mean Discrepancy before and after alignment as a distributional quality measure. The alignment itself is performed by standardizing the source variable and rescaling it toward the target mean and target standard deviation:

$$\mathrm{aligned}=\frac{\mathrm{source}-\mathrm{source}\_\mathrm{mean}}{\mathrm{source}\_\mathrm{std}}\times \mathrm{target}\_\mathrm{std}+\mathrm{target}\_\mathrm{mean}.$$

This operation is deliberately restricted to shared canonical columns, preventing inappropriate alignment between different physical quantities such as temperature and pressure.

The KPCA module provides nonlinear feature extraction through an RBF kernel. It first fills missing numerical values with medians, standardizes the data, and then applies KernelPCA. When the number of components is not explicitly specified, the prototype selects a conservative value based on the minimum of 8, the number of numerical columns, and the number of rows. This makes KPCA usable in small industrial batches while avoiding an excessively high-dimensional transformed space.

The LSTM temporal-correction module is implemented as an LSTM autoencoder. Before training, numerical columns are linearly interpolated and median-filled. The module then constructs sliding windows with a default window size of 10. The autoencoder consists of an input layer, an LSTM encoder with 64 hidden units, a repeat-vector layer, an LSTM decoder with 64 hidden units, and a time-distributed dense output layer. It is trained with the Adam optimizer, a learning rate of 0.001, and mean squared error loss for 100 epochs by default. After reconstruction, the module computes the reconstruction error for each window. Windows whose reconstruction error exceeds the mean error plus three standard deviations are treated as anomalous. For each anomalous window, the central time point is replaced by the reconstructed value, which allows local temporal correction without rewriting the entire sequence. If TensorFlow is unavailable or the dataset is too short for the selected window size, the module safely returns the original dataframe unchanged.

A key implementation principle is that the AI Agent never assumes a fixed pipeline. Instead, it invokes execution tools in different combinations according to the generated plan. For example, a dataset with mild missingness and a relatively stable distribution may only require KNN imputation followed by min-max normalization. A dataset with abnormal spikes and cross-source distribution mismatch may require IQR correction followed by MMD distribution alignment. A dataset with strong temporal instability may require LSTM-based temporal correction after basic cleaning. This modular architecture also reduces system-level overfitting. Because the framework is governance-centered, the numerical tools can be replaced or upgraded without changing the semantic catalog or decision layer. The implementation modules are shown in Table 3 below.

### 3.5. Implementation Procedure

The prototype implementation can be summarized in the following steps.

1. **Input construction.** Raw heterogeneous sensor streams are loaded into a pandas.DataFrame. Each column represents a raw sensor field. A metadata list is constructed using SensorMetadata, where each metadata record contains the original field name, mean value, unit, and sampling frequency.
2. **Catalog-based semantic audit.** The metadata list is passed to the catalog labeler. The labeler applies keyword-based, unit-based, value-range-based, and frequency-based rules to infer the likely sensor type of each field.
3. **Probabilistic semantic labeling.** If Snorkel is available, the labeling functions are applied through LFApplier, and a LabelModel is trained to infer probabilistic labels. If Snorkel is unavailable, a fallback voting strategy aggregates the labeling-function outputs. In both cases, the result contains the field name, predicted label, confidence score, and class-probability distribution.
4. **Canonical field mapping.** The pipeline converts predicted sensor types into canonical names such as temperature, pressure, vibration, current, and voltage. Duplicate canonical names are disambiguated with numerical suffixes. Low-confidence or unknown fields are preserved under their original names and flagged for later catalog enrichment.
5. **Data-state perception.** The aligned dataframe is analyzed by the perception module. The module computes the missing-value ratio, estimates the noise level from first-difference volatility, and detects whether any numeric column contains IQR-based outliers.
6. **Agent planning or heuristic planning.** The semantic map and data-state vector are combined into a planning prompt. If a LangChain-compatible LLM agent is available, the agent selects a processing strategy by reading the prompt and invoking registered tools. If no LLM is available, the deterministic heuristic planner generates the processing sequence according to explicit rules.
7. **Tool execution.** The selected tools are applied to the working dataframe. The currently implemented callable tools include KNN imputation, IQR anomaly correction, PCA dimensionality reduction, MMD distribution normalization, and LSTM temporal correction. Each tool reads the current working dataframe and writes the processed result back into working memory.
8. **Fallback processing.** If an exception occurs at any stage, the system switches to a robust fallback flow consisting of linear interpolation, median filling, and min-max normalization. This ensures that the pipeline returns a usable processed dataset rather than failing silently.
9. **Output and feedback.** The final output is a cleaned and normalized dataframe. In addition, the semantic map, data-state vector, selected tool sequence, and relevant algorithmic logs can be appended to the Intelligent Catalog as provenance records. These records support later audit, reproduction, and catalog evolution.

This implementation procedure makes the abstract “alignment—cleaning—normalization” workflow concrete and executable. It also clarifies that the AI Agent is not a black-box replacement for numerical methods. Rather, it acts as a governance-oriented workflow controller that decides which transparent numerical tools should be executed under which semantic and statistical conditions.

### 3.6. Algorithmic Description of the Proposed Method

To formalize the aforementioned system architecture and implementation steps, the data cleaning and normalization process orchestrated by the AI Agent is summarized as an algorithmic procedure. The procedure starts from the ingestion of multi-source heterogeneous sensor data and metadata, performs semantic alignment through the Intelligent Catalog, extracts data-state indicators, selects an execution strategy, invokes modular processing tools, and returns normalized data together with governance feedback. The implementation process is shown in the Table 4 below.

**Figure 2:**
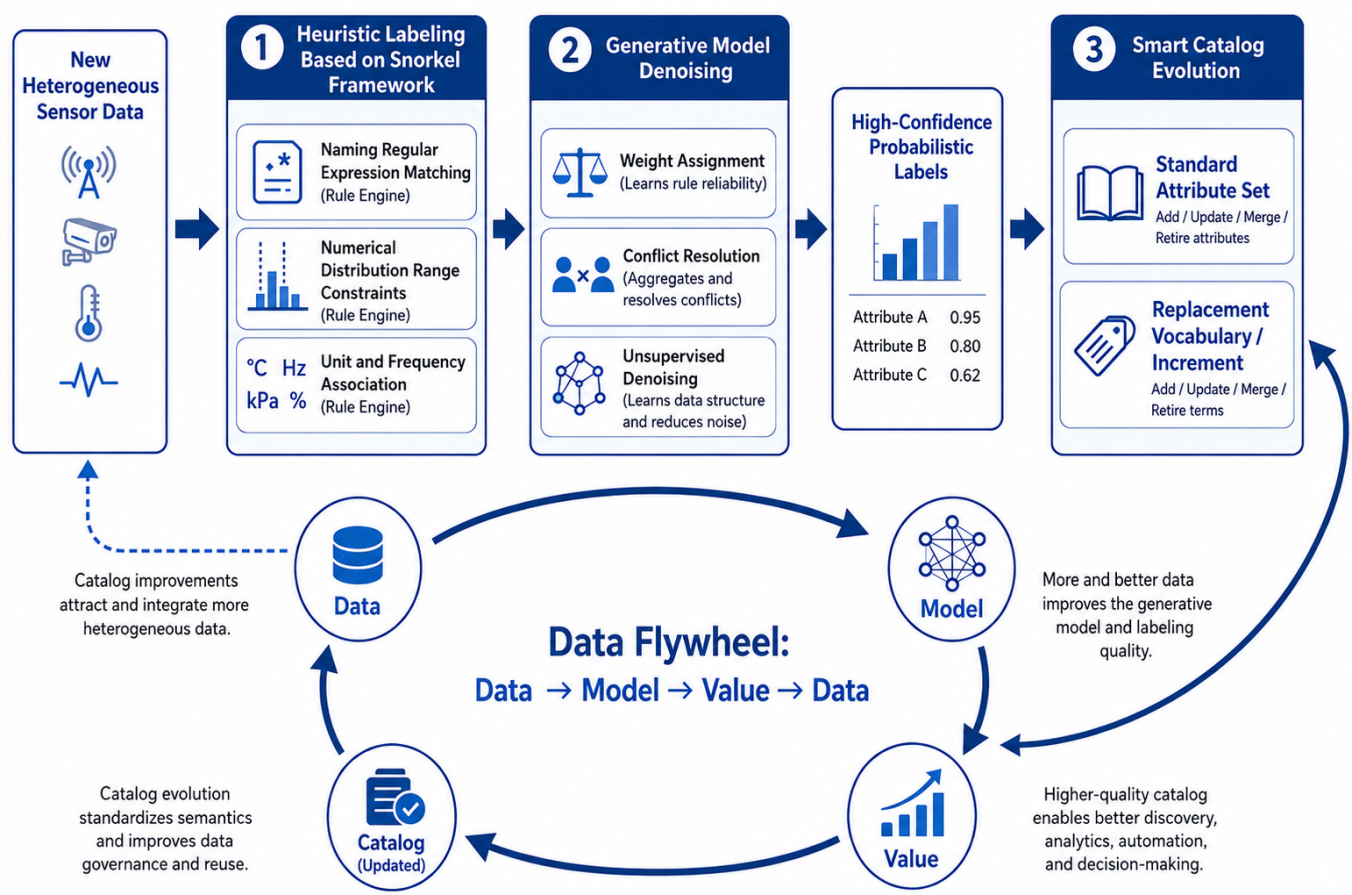
Dynamic catalog evolution mechanism based on data programming and a data flywheel. New heterogeneous sensor data are weakly labeled through heuristic rules, denoised by a generative model, converted into high-confidence probabilistic labels, and used to update the standard attribute set and replacement vocabulary.

The above algorithm corresponds to the flowchart shown in Figure 2. The input data first undergo semantic alignment, after which the state-vector extraction results are fed into the AI Agent’s decision logic matrix to trigger specific processing modules. These modules include missing-value imputation, denoising and outlier correction, distribution-aware normalization, nonlinear feature extraction, and temporal correction. The system finally produces three outputs: normalized data, provenance audit records, and feedback for iterative catalog updates.

### 3.7. Iterative Enhancement and Governance Feedback Mechanism

Industrial sensor systems continually change as new equipment, new sensor types, and new standards are introduced. Static catalogs cannot sufficiently support such changes. Therefore, the framework incorporates iterative governance through automatic annotation, weak supervision, and a data flywheel mechanism.

The proposed system uses data programming based on the Snorkel framework to generate high-quality training labels with weak supervision. Domain knowledge is encoded as labeling functions. In the current implementation, these labeling functions include naming-pattern matching, value-unit constraints, and sampling-frequency rules. For example, fields containing temp, temperature, wendu, or deg provide evidence for the temperature class. Fields containing press, pressure, or pa, combined with units such as mpa, bar, or kpa, provide evidence for the pressure class. Fields containing vib, vibration, or accel, together with a high sampling frequency, provide evidence for the vibration class. These functions may overlap or conflict, but their outputs are aggregated by the Snorkel label model into probabilistic labels. When Snorkel is unavailable, the fallback voting mechanism computes the dominant non-abstaining vote and assigns a confidence score based on the proportion of supporting labeling functions.

The catalog update follows a conservative governance principle. High-confidence labels are used to update the standard attribute set and attribute substitution vocabulary. Low-confidence or abstained cases are not forced into the catalog; instead, they are retained as candidates for expert review or future labeling-function refinement. This mechanism is consistent with the bibliographic principle of authority control: new variant names can be linked to existing standard headings when sufficient evidence exists, while uncertain expressions remain under review to avoid contaminating the controlled vocabulary.

The data flywheel provides a self-reinforcing closed loop. More heterogeneous data improve the coverage of labeling functions and probabilistic label quality. Improved labels update the catalog. A better catalog improves semantic alignment, data discovery, workflow automation, and downstream analytics. Higher-quality analytics, in turn, encourage further data integration and reuse. Through this loop, the Intelligent Catalog evolves from an initial expert-guided schema into a continuously enriched governance infrastructure.The relationship between the data flywheel and the catalog evolution mechanism is shown in Figure 2.

In the current prototype, the feedback mechanism is realized through four concrete artifacts: the semantic map generated during _semantic_align(), the confidence scores and probability distributions returned by generate_probabilistic_labels(), the data-state vector produced by perceive_data_state(), and the operation logs generated by processing modules such as MMD distribution normalization and LSTM temporal correction. In an industrial deployment, these artifacts can be stored as append-only catalog records, including source identifier, original field name, canonical attribute name, mapping confidence, selected tools, parameter settings, before/after distribution statistics, and timestamped execution records. This design makes the preprocessing procedure traceable, reproducible, and auditable.

**Figure 3:**
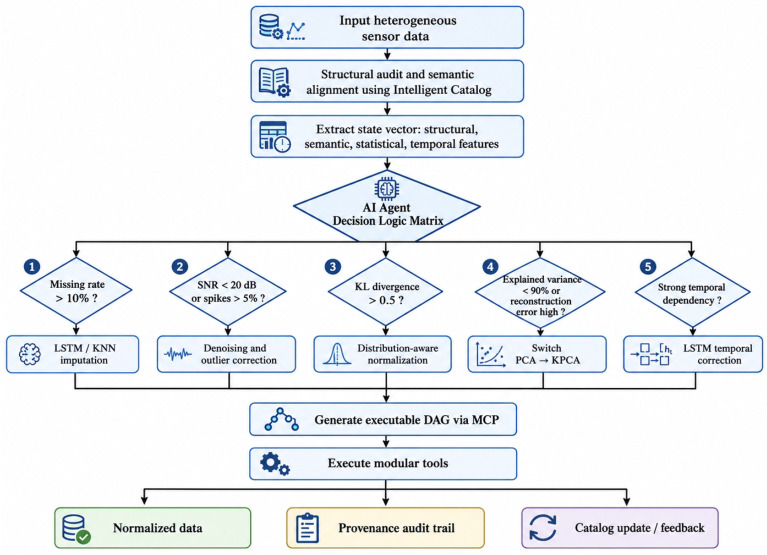
Flowchart of the AI Agent-orchestrated cleaning and normalization procedure. The figure shows semantic alignment, decision triggers, DAG generation, modular tool execution, and the outputs: normalized data, provenance records, and catalog feedback.

Overall, the proposed framework shifts industrial sensor-data cleaning and normalization from isolated algorithmic preprocessing to catalog-driven data governance. The Intelligent Catalog provides semantic authority control; the AI Agent provides adaptive workflow orchestration; and the execution layer provides transparent, replaceable numerical tools. This combination allows heterogeneous industrial sensor data to be cleaned, normalized, and reused in a manner that is both technically executable and consistent with information-governance principles.The overall structure is shown in the Figure 3 below.

## 4. Experimental Design and Setup

### 4.1. Experimental Objectives

The experimental evaluation systematically verifies whether the proposed governance-centered framework can effectively address the challenges of cleaning and normalizing heterogeneous industrial sensor data. The verification focuses on three core research questions:

- **RQ1**: Can the framework improve normalization consistency among heterogeneous data sources?
- **RQ2**: How robust is it under different noise levels, missing rates, and distribution change conditions?
- **RQ3**: Compared to algorithm-centered baseline methods, does the governance-centered design have advantages in stability and scalability?

The experimental design explicitly maps evaluation objectives to methodological claims, emphasizing validation of the entire framework rather than single algorithm components, while considering the practicality of industrial scenarios to ensure the results support engineering applications.

### 4.2. Dataset Description and Heterogeneity Characterization

The experiments utilize two types of datasets for general performance verification and industrial case verification, both containing heterogenous features and quality issues of real industrial sensor data.

#### 4.2.1. General Public Datasets

Three typical industrial sensor datasets were selected from *awesome-industrial-datasets*, covering chemical processing, smart manufacturing, and energy monitoring as shown in Table 5 below.

#### 4.2.2. Industrial Case Dataset

The *Industrial IoT Fault Detection Dataset* was selected for industrial case verification [19]. Sourced from a real IIoT equipment monitoring scenario, it is widely used in condition monitoring and fault detection research, providing significant engineering representation.

- **Multi-Sensor Heterogeneity**: Contains five types of sensors (temperature, pressure, vibration, current, voltage) across 30 heterogeneous sensor nodes, with clear differences in dimensionality, value ranges, sampling frequencies (1–10 Hz), and noise characteristics.
- **Real Industrial Quality Issues**: Prevalent measurement noise (Gaussian, impulse), abnormal spikes, and intermittent missing values, simulating real scenarios like sensor aging, communication packet loss, and industrial interference.
- **Operating Condition Diversity**: Includes normal operation and five typical fault conditions (bearing wear, circuit overload, temperature anomalies, etc.), providing ideal testing conditions for evaluating data cleaning, normalization, and downstream fault detection.

#### 4.2.3. Heterogeneity Characterization

For all experimental datasets, heterogeneity is characterized across structural, semantic, and statistical dimensions to clarify the core difficulties of data governance. And these are showen in Table 6 below.

### 4.3. Baseline Methods and Configurations

To comprehensively verify the performance advantages of the proposed framework, five representative methods from different paradigms were selected as baselines. All baseline methods were configured according to best practices in the literature and run under the same data splitting (70% training, 30% testing) and hardware environment to ensure fairness. And these are shown in Table 7 below.

### 4.4. Evaluation Metrics

Combining experimental objectives with industrial data governance needs, four complementary metrics were adopted to comprehensively measure the framework’s performance:

It is worth noting that Normalization Consistency (NC) is not merely a statistical measure, but a critical safeguard for physical semantic integrity. High stability post-normalization is paramount because it ensures that the framework reliably eliminates technical bias (e.g., sensor drift) without arbitrarily erasing actual physical variations (such as the spatial gradients discussed in Section 5.1). If a normalization method is unstable, it risks “over-normalizing” the data—destroying the true spatial and contextual differences—which would inevitably lead to false positives or missed detections in downstream industrial applications (e.g., fault diagnosis). Therefore, a high and stable NC score directly correlates to the reliability of the data for subsequent intelligent decision-making.

To ensure clarity and reproducibility, we specifically define the components of the Anomaly Detection Accuracy (ADA) metric. In this study, a “correctly identified anomaly” (True Positive, TP) is defined at the point-wise level: it refers to a specific temporal data point that is labeled as noise, missing, or an outlier in the ground-truth dataset, and is successfully flagged by the framework’s anomaly initial screening or LSTM modules prior to normalization. Conversely, “correctly identified normal data” (True Negative, TN) refers to a clean data point that the system correctly leaves unaltered. The ADA metric represents the strict point-to-point classification accuracy (TP+TN)/Total, evaluating the AI Agent’s precision in applying targeted cleaning strategies without disturbing healthy data.And the details are shown in the Table 8 below.

### 4.5. Experimental Environment

All experiments were run under a unified hardware and software environment to ensure reproducibility:

- **Hardware**: Intel Core i7-12700H CPU (14 cores, 20 threads), 32 GB RAM, 1 TB SSD, NVIDIA RTX 3060 GPU (6 GB VRAM).
- **Software**: Windows 11 Professional, Python 3.10, TensorFlow 2.8.0, Pandas 1.4.2, NumPy 1.21.5. Intelligent Catalog built using Snorkel framework 0.19.0; AI Agent implemented using LangChain 0.0.200 combined with the MCP protocol, powered by the gpt-4-turbo large language model to execute the core reasoning and orchestration tasks.

## 5. Industrial Case Verification

Based on the Industrial IoT Fault Detection Dataset, we conducted an industrial case verification to further validate the framework’s effectiveness and practical value in real industrial scenarios, fully reproducing the “semantic alignment—intelligent decision—numerical processing” workflow.

### 5.1. Case Dataset Preprocessing and Intelligent Catalog Construction

Targeting the dataset’s heterogeneous features, an Intelligent Catalog tailored to this industrial scenario was first constructed to achieve semantic governance and preliminary alignment:

- **Standard Attribute Set Construction**: Defined five categories of standard attributes covering sensor functional types (thermal, mechanical, electrical), measured physical quantities, engineering units, expected value ranges, and sampling frequencies to cover core semantic info.
- **Attribute Substitution & Semantic Alignment**: Constructed the attribute substitution table to map heterogeneous names to standard attributes. Three ambiguous fields that could not be automatically matched were flagged as “low-confidence semantic items” for subsequent correction via the governance feedback mechanism.
- **Dynamic Catalog Updates**: Based on the Snorkel framework and Data Flywheel, new data features were automatically annotated, adding metadata for two new sensor types (humidity, rotational speed) to the standard attribute set, realizing dynamic catalog evolution.

In this case study, we specifically modeled the spatial relationship between the 30 heterogeneous sensor nodes. By labeling sensors based on their thermal and mechanical proximity in the Intelligent Catalog, we enabled the AI Agent to identify that certain temperature variances were caused by spatial heat gradients rather than sensor malfunctions, thereby avoiding erroneous over-normalization.

### 5.2. AI Agent Strategy Selection and Workflow Orchestration

After semantic alignment, the AI Agent performed multi-dimensional data status perception, identifying core features: most sensors had moderate noise (20–25 dB SNR), 10 sensors had intermittent missing values (5–15%), significant statistical distribution differences existed (KL mean 0.82), and data fluctuation amplitudes increased by >30% under fault states.

Based on these features, the AI Agent dynamically selected multi-stage data governance strategies and orchestrated the following workflow.

1. **Anomaly Initial Screening and Noise Mitigation**: Used IQR (1.5×) to identify outliers, smoothed abnormal spikes, and applied median filtering to mitigate Gaussian and impulse noise.
2. **Missing Value Priority Correction**: Used LSTM models for sensors with ≥10% missing data; used KNN (k = 5) for <0%, ensuring completeness.
3. **Distribution-Aware Normalization**: Used MMD to quantify distribution differences across sensors and adaptively scaled the data to achieve cross-sensor distribution alignment. It is critical to clarify the foundational assumption of this step: distribution alignment is strictly restricted to homologous sensors—sensors that are semantically verified by the Intelligent Catalog to measure the exact same physical quantity under comparable operational contexts. The AI Agent never attempts to scale or align disparate physical modalities (e.g., temperature and pressure).
  Furthermore, to address potential concerns regarding the loss of metrological traceability, this scaling is designed as a non-destructive, parallel transformation solely intended to optimize the convergence of downstream machine learning models (e.g., the LSTM classifier). The raw metrological data is never overwritten. The AI Agent rigorously logs all scaling parameters (e.g., original minimum, maximum, mean, and variance values) into the provenance audit trail. This guarantees that any normalized value fed into the neural network can be mathematically inverted to retrieve its exact, original physical unit and absolute measurement state, thereby fully preserving metrological traceability.
4. **Temporal Auxiliary Correction**: For highly time-dependent sensors (vibration, current), invoked LSTM models to capture temporal features and apply secondary corrections to abnormal fluctuations, enhancing data stability.

### 5.3. Case Experiment Results and Analysis

The proposed framework was compared against four baseline methods on the case dataset, evaluating normalization consistency, robustness, and impact on downstream fault detection:

#### 5.3.1. Normalization Consistency Analysis

The proposed framework’s NC value reached 0.92, significantly outperforming all baseline methods. It showed an 18.9% improvement over the best-performing KPCA method and a 43.8% improvement over traditional min-max normalization, indicating the framework effectively improves NC through semantic alignment and distribution-aware normalization, successfully answering RQ1.And the details are shown in Table 9 below.

#### 5.3.2. Robustness Analysis

Robustness was tested by incrementally increasing noise (SNR from 30 dB down to 5 dB) and missing ratios (from 5 to 40%):

- **Noise Robustness**: At 10 dB (high noise), the framework’s NC value remained at 0.78 (only a 15.2% drop). KPCA’s NC dropped to 0.59 (23.4% drop), and min-max normalization dropped to 0.41 (35.9% drop), demonstrating stronger adaptability to industrial noise due to the AI Agent’s dynamic strategy adjustment.
- **Missing Value Robustness**: At a 40% missing ratio, the framework’s NC was 0.71 (22.8% drop). The best baseline (KPCA) dropped to 0.53 (31.2% drop), and traditional methods dropped below 0.45, showing the effectiveness of the adaptive correction strategy.

These results indicate good robustness under varying noise and missing rate conditions, addressing RQ2.

#### 5.3.3. Impact on Downstream Fault Detection

Using the same fault detection model (LSTM classifier), data preprocessed by the proposed framework achieved a 94.7% fault detection accuracy, an 8.3% improvement over KPCA and a 15.6% improvement over min-max normalization. This demonstrates that high-quality data governance directly enhances downstream intelligent application performance.

#### 5.3.4. Robustness Against Semantic Shift and Structural Heterogeneity

To further validate the necessity of a governance-centered approach in real-world IIoT environments, we introduced a structural interference test. In this test, 20% of the sensor column names in the test set were randomly modified (e.g., renaming “voltage_1” to “V_in”), and a random subset of sensor input dimensions was dropped.

Under these conditions, algorithm-centered models like LSTM-AE and PatchTST experienced catastrophic failures or severe performance degradation due to input shape mismatch and unresolvable column indices. In contrast, the proposed framework maintained stable operation. Leveraging the Intelligent Catalog, the AI Agent automatically re-established semantic mappings via zero-shot dynamic adaptation, sustaining high Alignment Consistency (AC). This explicitly proves the framework’s superior fault tolerance and zero-maintenance capability in dynamically evolving industrial deployments.

## 6. Ablation Study

To verify the necessity and synergistic effect of the framework’s core components (Intelligent Catalog, AI Agent, Governance Feedback Mechanism), an ablation study was designed by removing different components to build four contrast versions. All versions used the same downstream model and configurations. And the details are shown in Table 10 below.

### 6.1. Ablation Study Settings

- **Full Framework**: Contains all layers and mechanisms.
- **Catalog**: Removes the Intelligent Catalog layer; raw heterogeneous data is processed numerically without semantic alignment.
- **Agent**: Removes the AI Agent decision layer; uses a fixed processing flow without adaptive strategy selection.
- **Feedback**: Removes the dynamic catalog update mechanism; the standard attribute set and substitution table remain static.

### 6.2. Ablation Study Results and Analysis

- **Role of Intelligent Catalog**: Removing it caused the most significant NC drop (from 0.92 to 0.67, 27.2% decrease) and an AC drop of 21.7%. This confirms semantic alignment is vital; without it, numerical normalization loses unified reference, causing “numerical alignment but semantic misalignment”.
- **Role of AI Agent**: Removing it caused NC to drop to 0.78 (15.2% decrease) and ADA to drop to 0.85 (8.6% decrease). Fixed flows cannot adapt to varying data states, highlighting the AI Agent’s necessity for robustness.
- **Role of Feedback Mechanism**: Removing it caused slight drops (NC down 7.6%, ADA down 3.2%), indicating dynamic updates improve adaptability to new data.
- **Synergy**: The full framework achieved optimal performance across all metrics with only slightly higher computational overhead, proving all components are synergistic and non-redundant.

## 7. Sensitivity and Scalability Analysis

### 7.1. Sensitivity Analysis

To further answer RQ2, we evaluated the framework’s stability under different noise levels and missing proportions.

- **Sensitivity to Noise**: As SNR dropped from 30 dB to 5 dB, the framework’s NC dropped from 0.95 to 0.75 (21.1% decline). KPCA-LSTM dropped by 37.8%, and min-max normalization dropped by 45.7%, showing lower sensitivity to noise.
- **Sensitivity to Missing Data**: As the missing ratio increased from 5% to 40%, the framework’s NC dropped from 0.94 to 0.71 (24.5% decline). KPCA-LSTM dropped by 38.3%, and rule-based methods dropped by 50.8%. The AI Agent effectively limits performance degradation.

### 7.2. Scalability Analysis and Large-Scale Verification

To evaluate the framework’s performance under realistic large-scale IIoT deployments, we extended the scalability analysis from 200 to 500 heterogeneous sensor nodes. To overcome the limitations of the original dataset scale, we utilized robust data augmentation techniques—including time-series shifting, Gaussian noise injection, and metadata semantic permutation—to synthesize a large-scale dataset comprising 6.5 million time-series records.

The evaluation focused on system throughput, processing latency, and alignment robustness at this expanded scale.

For computational overhead, as the node count increased to 500, the framework’s throughput dropped to approximately 3800 records/s, and the latency increased to 48.5 s per 100k records. This is significantly higher than the traditional KPCA-LSTM baseline (which maintained 6500 records/s). This performance degradation is primarily attributed to the increased computational burden of the AI Agent concurrently orchestrating multiple complex DAG workflows and the LLM API overhead.

For robustness at-scale, despite the higher latency, the governance-centered design demonstrated overwhelming superiority in alignment stability. Under the extreme heterogeneous conditions of 500 nodes, the traditional KPCA-LSTM model suffered a severe performance collapse, with its Normalization Consistency (NC) dropping to 0.61 due to input dimension mismatch and semantic drift. In stark contrast, our proposed framework maintained an NC of 0.87 and an Alignment Consistency (AC) of 0.89.

For analysis, these results indicate that while the AI Agent introduces inevitable computational bottlenecks for strict real-time (millisecond-level) processing at scale, it provides an unparalleled fault tolerance and semantic alignment capability for large-scale, offline, or near-real-time IIoT data governance scenarios.

### 7.3. Lifecycle Computational Overhead Under Data Drift

While pure deep learning models like LSTM-AE exhibit extremely fast forward inference speeds (e.g., processing 100k records in approximately 2 s), this metric only reflects ideal, static conditions. In practical industrial scenarios, the frequent occurrence of data drift and sensor updates renders static models brittle.

When structural or distribution shifts occur, maintaining deep learning models requires collecting tens of thousands of new data points and spending hours or even days on model retraining. Conversely, the proposed AI Agent framework handles these heterogeneous shifts by automatically updating a few metadata tags within the Intelligent Catalog, a process that takes only a few minutes. Therefore, from a system-level lifecycle perspective, the governance-centered framework incurs significantly lower long-term operational and maintenance overhead compared to pure deep learning approaches.

## 8. Overall Experimental Results and Conclusions

### 8.1. Overall Performance Comparison

Across all datasets, the proposed framework outperformed rule-based and pure statistical methods in all metrics. Compared to learning-based baselines, it matched or exceeded them in NC and AC with significantly lower performance fluctuations under varying conditions, though with higher time overhead.

- **NC**: Averaged 0.90 (16.7% improvement over KPCA-LSTM).
- **ADA**: Averaged 0.92 (7.1% improvement over KPCA-LSTM).
- **AC**: Averaged 0.91 (14.8% improvement over KPCA-LSTM).
- **CO**: Averaged 11.8 s/100k records, only slightly lower than complex deep learning but 32.6% higher than KPCA-LSTM, highlighting its primary shortcoming.

### 8.2. Experimental Conclusions

Based on the results, we can comprehensively answer the three research questions:

- **RQ1**: The framework significantly improves normalization consistency among heterogeneous sources, resolving the “numerical alignment but semantic misalignment” issue.
- **RQ2**: The framework demonstrates good robustness and lower sensitivity under various noise levels and missing rate conditions.
- **RQ3**: The governance-centered design offers significant stability advantages over algorithm-centered baselines. However, the time overhead limits its scalability and feasibility for high real-time demand, complex industrial environments.

Furthermore, ablation studies validated the necessity of all core components, and the industrial case proved its engineering utility, providing technical support for future IIoT data governance platforms.

## 9. Limitations and Future Work

Despite its advantages, the system has several limitations. The initial construction of the attribute and substitution tables relies on domain knowledge, limiting scalability. The AI Agent, which is primarily prompt-based, performs worse than fully fine-tuned LLMs. Given the size of LLMs and the system’s overall slow execution speed, it is currently difficult to apply it directly to industrial production.

Given these issues, future work will introduce a “Separation of Control Plane and Data Plane” design pattern from distributed systems to build an “offline orchestration—online execution” dual-state architecture. The specific evolution idea is as follows:

- **Offline Control Plane**: The AI Agent is removed from the real-time critical path, relying on historical or micro-batch data to perform complex offline matching and decision-making, compiling the workflow into a static Directed Acyclic Graph (DAG) configuration to be sent to the data plane.
- **Online Data Plane**: Lightweight execution agents deployed on edge nodes or stream engines execute the DAG using underlying Rust-based algorithm libraries for millisecond-level “blind execution,” bypassing LLM overhead and vastly improving throughput.
- **Data Drift Closed-Loop Mechanism**: Lightweight data drift monitoring will be introduced online. When significant statistical distribution deviations occur, it triggers an alarm to wake the offline AI Agent for re-evaluation and DAG regeneration, balancing system performance and real-time capability.

## Figures and Tables

**Table 1:** Improved system architecture description.

Layer	Primary Responsibility	Typical Inputs	Typical Outputs	Representative Components
Intelligent Catalog Layer	Semantic governance, metadata standardization, field alignment, controlled vocabulary management	Raw metadata, heterogeneous schema, naming variants, units, sampling frequency	Standardized attributes, mapping confidence, semantic map, catalog records	Standard attribute set; attribute substitution table; semantic labeling functions; probabilistic label model
AI Agent Decision Layer	State perception, strategy selection, workflow orchestration, provenance planning	Semantic map; missing ratio; noise level; outlier status; optional target-domain information	Governance policy, execution sequence, decision trace, executable tool plan	Data-state perception module; LangChain-compatible agent; heuristic fallback planner; working-memory manager
Execution Layer	Data cleaning, missing-value imputation, outlier correction, dimensionality reduction, distribution alignment, temporal correction, normalization	Executable plan; aligned numerical streams; optional target dataframe	Normalized data, corrected data, transformed features, scaling parameters, operation logs	IQR correction; KNN imputation; PCA; KPCA; RBF-MMD normalization; LSTM autoencoder; min-max normalization

**Table 2:** Decision indicators and prototype trigger rules for the AI Agent.

Indicator	Methodological Meaning	Prototype Operationalization	Selected Strategy	Rationale
Missingness	Measures structural incompleteness of the data table	missing_ratio = number of NaN cells/total cells; if greater than zero in the current fallback planner	KNN imputation	Missing values should be repaired before scaling, dimensionality reduction, or distribution alignment
Noise severity	Measures short-term signal instability	First-difference volatility ratio; score <0.5 = low, 0.5–1.2 = medium, >1.2 = high	IQR anomaly correction; LSTM correction when high	Prevents spikes and unstable fluctuations from distorting normalization
Outlier status	Identifies extreme values in numeric streams	IQR rule: values outside [Q1−1.5×IQR,Q3+1.5×IQR]	IQR clipping/ correction	Controls pulse-like industrial interference while preserving the median trend
Cross-source divergence	Measures distribution mismatch among semantically homologous fields	Optional target dataframe; shared canonical columns are aligned through RBF-MMD-guided normalization	Distribution-aware normalization	Aligns comparable sources while preserving semantic consistency
Nonlinear structure	Captures nonlinear feature relationships	KPCA module available in the execution layer with RBF kernel	KPCA feature extraction	Preserves nonlinear structures in heterogeneous sensor data
Temporal dependence	Captures sequential patterns and anomalous temporal windows	LSTM autoencoder branch activated under high noise or strong temporal-dependency conditions	LSTM temporal correction	Uses reconstruction error to identify and correct temporally abnormal segments

**Table 3:** Prototype implementation modules.

Module	Function	Concrete Implementation
Metadata schema	Represents raw sensor metadata and standard sensor types	SensorMetadata dataclass; SensorType enumeration
Catalog labeler	Generates probabilistic semantic labels for raw metadata	Snorkel labeling functions; fallback voting
Semantic alignment pipeline	Converts raw column names into canonical names	_semantic_align() in IndustrialDataGovernancePipeline
Data-state perception	Computes executable indicators for agent planning	Missing ratio; first-difference volatility; IQR outlier flag
AI Agent planner	Selects and chains tools	LangChain agent when available; heuristic fallback otherwise
Tool registry	Provides callable processing modules	LangChain-compatible wrappers in agent/tools.py
Basic processing toolkit	Performs imputation, outlier correction, PCA, and normalization	IQR clipping; KNN; PCA; min-max scaling
Advanced processing toolkit	Performs distribution and temporal correction	RBF-MMD alignment; RBF-KPCA; LSTM autoencoder
Fallback processor	Ensures runtime robustness	Linear interpolation; median filling; min-max scaling
Provenance support	Records key transformation states	Semantic map, data state, selected steps, logging of MMD and LSTM events

**Table 4:** AI Agent-orchestrated cleaning and normalization procedure.

Step	Operation
1	Input raw sensor dataframe *D*, metadata records *M*, and optional target dataframe *T*.
2	Register metadata records in the Intelligent Catalog using the standard metadata schema.
3	Apply semantic labeling functions to each metadata record, using keyword, unit, value-range, and sampling-frequency evidence.
4	Generate probabilistic semantic labels through the Snorkel label model or fallback voting mechanism.
5	Construct a semantic mapping from raw field names to canonical attribute names. If confidence is insufficient, retain the original name and mark the item for catalog review.
6	Rename the raw dataframe according to the semantic mapping to obtain the semantically aligned dataframe $D_{\mathrm{align}}$.
7	Compute the data-state vector *s*, including missing ratio, noise level, and IQR-based outlier status.
8	Build a planning prompt from the semantic map and data-state vector.
9	If a LangChain-compatible LLM agent is available, initialize the governance agent and let it select the processing strategy.
10	If the LLM agent is unavailable, use the deterministic heuristic strategy planner.
11	If missing values are detected, add KNN imputation to the workflow.
12	If outliers are detected or the noise level is medium/high, add IQR anomaly correction to the workflow.
13	If a target dataframe is available for homologous variables, add RBF-MMD distribution normalization to the workflow.
14	If the noise level is high or strong temporal instability is observed, add LSTM temporal correction to the workflow.
15	Execute the selected tools sequentially on the working dataframe.
16	If nonlinear feature extraction is required, apply RBF-KPCA through the same standardized execution interface.
17	If any stage fails, execute the fallback flow: linear interpolation, median filling, and min-max normalization.
18	Return the processed dataframe $D^{*}$, semantic map, selected operation sequence, processing parameters, and governance feedback.
19	Append high-confidence mappings and execution traces to the Intelligent Catalog for future reuse.

**Table 5:** Awesome-industrial-datasets metadata.

Dataset Name	Sensor Types	Data Scale	Core Features
Chemical Process Sensor Dataset	Temperature, pressure, flow	1 M + time-series records, 20 heterogeneous sensors	Sensor drift, inconsistent semantic naming, significant statistical distribution differences
Smart Factory Sensor Dataset	Vibration, current, voltage	800 k + time-series records, 15 heterogeneous sensors	Intermittent missing values, industrial electromagnetic noise, obvious nested structure differences
Energy Grid Sensor Dataset	Power, frequency, voltage	1.2 M + time-series records, 25 heterogeneous sensors	Multi-source semantic overlap, inconsistent sampling frequencies, prominent non-stationary features

**Table 6:** Types and manifestations of heterogeneity.

Heterogeneity Type	Specific Differences	Typical Manifestations
Structural	Differences in data schemas, nested levels, and formats	Flat tables vs. nested JSON; inconsistent sampling frequencies (fixed intervals vs. irregular sampling)
Semantic	Inconsistent field naming and attribute semantic overlap	“Temperature” labeled as “Temp”, “Temperature”, “Wendu”; similar field names with different meanings (e.g., “Current 1” referring to different circuits)
Statistical	Value ranges, distribution shapes, and noise modes	Temperature ranges −20∼150 °C vs. pressure 0∼10 MPa; normal vs. skewed distributions; Gaussian vs. impulse noise

**Table 7:** Baseline methods and parameter configurations.

Baseline Type	Specific Method	Parameter Configuration
Traditional Rule-Based	Expert Rule-based Cleaning and Normalization	Outlier threshold (±3σ), mean imputation for missing values, fixed ratio scaling
Statistical Normalization	Min–Max Normalization	Normalization range [0, 1], outliers truncated
Statistical Normalization	Z-Score Standardization	Mean μ = 0, std dev σ = 1, outliers retain original distribution
Machine Learning	PCA-based Preprocessing	Retain 95% variance contribution, KNN imputation (k = 5)
Mixed Machine Learning	KPCA–LSTM Mixed Method	KPCA kernel = RBF, LSTM hidden nodes = 64, epochs = 100, learning rate = 0.001
Deep Learning	LSTM-Autoencoder (LSTM-AE)	Encoder/Decoder with 2 LSTM layers (64, 32 nodes), MSE loss function, anomaly determined by reconstruction error threshold
Deep Learning	Time-Series Transformer (PatchTST)	Multi-Head Attention (4 heads), Patch size = 16, 2 Encoder blocks. Optimized for capturing complex long-range dependencies

**Table 8:** Experimental evaluation metrics definition and calculation.

Metric Name	Abbr.	Definition	Calculation Method
Normalization Consistency	NC	Measures the degree of consistency in distributions after normalization; closer to 1 is better.	Calculates mean distribution similarity of normalized data using KL divergence. NC = 1 − (Average KL/Max KL)
Anomaly Detection Accuracy	ADA	Detects the platform’s robustness in handling outliers; closer to 1 is better.	ADA = (Correctly detected anomalies + Correctly identified normal data)/Total data volume
Alignment Consistency	AC	Evaluates overall consistency after semantic and numerical processing.	AC = 0.6 × NC + 0.4 × Semantic Alignment Accuracy (correctly mapped fields/total fields)
Computational Overhead	CO	Measures processing time and resource consumption; lower is better.	Total time (in seconds) for cleaning and normalization per batch of 100,000 records. Records CPU usage (average ≤80% is acceptable).

**Table 9:** Normalization consistency comparison on case dataset.

Processing Method	NC Value	Gap Compared to Proposed Framework
Proposed Framework	0.92	—
PatchTST (Transformer)	0.88	−4.3%
LSTM-AE	0.86	−6.5%
KPCA Method	0.77	−16.3%
PCA Method	0.72	−21.7%
Z-score Standardization	0.68	−26.1%
Min–Max Normalization	0.64	−30.4%

**Table 10:** Ablation study results comparison.

Contrast Version	NC	ADA	AC	CO (s/100k Records)
Full Framework	0.92	0.93	0.92	11.7
Catalog	0.67	0.81	0.72	10.9
Agent	0.78	0.85	0.80	11.2
Feedback	0.85	0.90	0.87	11.5

## Data Availability

All codes and data can be obtained from the following GitHub repository https://github.com/qqjx/sensors.git (accessed on 24 May 2026).
